# *SOX2* mediates cisplatin resistance in small‐cell lung cancer with downregulated expression of hsa‐miR‐340‐5p

**DOI:** 10.1002/mgg3.1195

**Published:** 2020-03-04

**Authors:** Fei Cui, Zhe‐xue Hao, Jin Li, Ya‐lei Zhang, Xu‐kai Li, Jian‐xing He

**Affiliations:** ^1^ Department of Cardiothoracic Surgery First Affiliated Hospital of Guangzhou Medical University Guangzhou China

**Keywords:** cisplatin resistance, miRNA, small‐cell lung cancer, *SOX2*

## Abstract

**Background:**

This study is aimed to unravel the genetic factors associated with microRNA (miRNA) expression in regulating sex‐determining region Y‐box 2 (*SOX2*)‐mediated cisplatin resistance in small‐cell lung cancer (SCLC).

**Methods:**

The relevance of *SOX2* expression in SCLC was analyzed in a panel of SCLC cells by quantitative real‐time PCR (qPCR) and western blot (WB). We selected DMS114 cell line, in which *SOX2* was amplified via lentiviral vector‐mediated transfection of the *SOX2* genes and tested for the half‐maximal inhibitory concentration (IC_50_) by MTS assay. High‐throughput sequencing and screening of differentially expressed miRNAs between *SOX2*‐overexpressing and normal control cells were performed. Finally, miRanda software was used to verify the miRNAs bound with *SOX2* and qPCR was used to identify the expression of miRNAs which were binding with *SOX2*.

**Results:**

Cisplatin‐resistant *SOX2*‐overexpressing DMS114 cell lines were successfully developed, showing a statistically significant increase in *SOX2* expression by qPCR and WB. Our results showed a typically higher IC_50_ value in *SOX2*‐overexpressing cells compared with the negative controls. The high‐throughput sequencing analysis revealed that 68 miRNAs were upregulated and 24 miRNAs were downregulated in the *SOX2*‐overexpressing cells. The 24 downregulated miRNAs were further verified. Of them, a cancer‐related miRNA, hsa‐miR‐340‐5p, showed a higher binding affinity with *SOX2* in network regulation mapping, which was also found to be markedly downregulated under qPCR analysis.

**Conclusion:**

We demonstrated that downregulated expression of hsa‐miR‐340‐5p may affect cisplatin resistance by mediating *SOX2* expression in SCLC cells, which may provide a potential target for the therapy of chemoresistant SCLCs.

## Graphical abstract

In this study, we aimed to unravel the genetic factors associated with miRNA expression in regulating the *SOX2‐*mediated cisplatin resistance in SCLC. Cisplatin‐resistant *SOX2* overexpressing DMS114 cell lines were successfully developed, which showed a statistically significant increase in *SOX2* expression by qPCR and western blot.

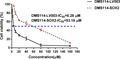

## 1 INTRODUCTION

Small‐cell lung cancer (SCLC) is a notoriously aggressive malignancy characterized by unique clinical features, such as rapid proliferative growth, early metastatic spread, and widespread dissemination (Tartarone et al., 2017). The incidence and mortality of SCLC worldwide represents 10%–15% of all lung cancers, and the lack of an effective drug for therapeutic intervention makes this disease a major public health problem (Altan & Chiang, 2015). The recommended standard first‐line SCLC treatment includes platinum‐based chemotherapeutics, like cisplatin (*cis*‐diamminedichloroplatinum) or carboplatin in combination with etoposide, cyclophosphamide, vincristine, or doxorubicin, which leads to complete remission in the vast majority of patients. Although SCLCs respond well to the initial chemotherapy (Singhal et al., 2006; Tripathi et al., 2017), relapse to treatment occurs within a year in SCLC patients and exhibits resistance to multiple drugs, with relapse and treatment resistance eventually strongly contributing to poor prognosis. Thus, SCLC remains a major therapeutic challenge for oncologists (Tartarone et al., 2017). The development of resistance toward chemotherapeutic drugs and a poor prognosis with a median survival of <1 year in SCLC patients with extensive disease make it a notable healthcare issue (Karachaliou, Rosell, & Viteri, 2013).

Cisplatin is a DNA‐damaging cytotoxic drug recommended as first‐line therapy in both SCLC and non‐small cell lung cancer (NSCLC), as well an effective anticancer drug to treat many other cancers (Lee et al., 2019). Recently, some reported evidence pointing toward the development of cisplatin resistance and the failure of treatment among cancer patients has questioned the efficacy of this drug (Galluzzi et al., 2014). Although many studies have been carried out to decipher the mechanism of the development of drug resistance to cisplatin, the exact mechanisms involved are not fully elucidated (Davidoff, Tang, Seal, & Edelman, 2010; Lee et al., 2002). Previously established mechanisms of cisplatin chemoresistance in cancer include cellular pathways associated with DNA damage and repair, apoptosis, NOTCH signaling, and FGFR signaling (Rodriguez‐Nieto & Zhivotovsky, 2006; Zhivotovsky, 2003).

Currently, advanced molecular studies have revealed that several cancers arise and progress via an accumulation of complex genetic and molecular alterations, which has enabled a better understanding of the tumor biology of clinically distinct tumor subclasses and has provided new diagnostic markers and potential targets for anticancer therapies (Wilbertz et al., 2011). Recent data have shown that several linage‐specific oncogenes are frequently amplified in significant subtypes of lung cancers, such as *NKX2‐1* (OMIM: 600635) in lung adenocarcinoma and amplification of the genomic region 3q in lung squamous cell carcinoma (SCC) (Barletta et al., 2009; Qian & Massion, 2008; Weir et al., 2007). Furthermore, the gene for a transcription factor, sex‐determining region Y‐box 2 (*SOX2*) was identified as the most promising candidate disease‐related gene associated with lung cancers (Bass et al., 2009; Hussenet et al., 2010).

*SOX2* (OMIM: 184429) is a member of the sex‐determining region Y‐chromosome‐related high mobility group‐box (SOX) family of transcription factors. It is expressed during early embryogenesis and plays an important role in embryonic and extra‐embryonic cell types (Avilion et al., 2003; Kamachi, Uchikawa, & Kondoh, 2000). Previous study of >50 tumor samples and SCLC cell lines showed that *SOX2* was amplified in approximately 27% of cancers (Rudin et al., 2012). Additionally, as SCLCs are tumors that possess neuroendocrine features, conditional induction of *SOX2* in lung epithelial cells is also known to increase the number of neural progenitor cells (Gontan et al., 2008), *SOX2* protein overexpression has also been previously noted in high‐grade SCLC, and immunoreactive antibodies against *SOX2* have been detected in sera from SCLC patients (Gure et al., 2000; Sholl, Long, & Hornick, 2010). The previous study has verified that *SOX2* gene overexpression inhibited cisplatin‐induced cell apoptosis in lung cancer cells, while *SOX2* knockdown led to an enhanced susceptibility of A549 and A549/DDP cells to cisplatin along with increased cell apoptosis (He et al., 2017), which suggested that *SOX2* may be an important regulator in chemoresistance of cisplatin in lung cancer cells. Thus, further studies should be conducted to determine if other molecules exist that affect *SOX2* expression in cisplatin‐resistant lung cancer cells.

Recent attention has focused on microRNAs (miRNAs) as potentially important upstream regulators in the development of chemoresistance (Lee et al., 2019), and accumulating data indicate that cisplatin resistance involves other pathways that are also modified by miRNAs (Lee et al., 2019; Xie et al., 2019; Zhang et al., 2018). In recent years, an increasing number of oncogenic and tumor‐suppressive miRNAs have been discovered in NSCLC (Guan, Yin, Li, Wu, & Zhou, 2012), such as miR‐625 (Li, Liang, & Zheng, 2018), miR‐15b (Wang, Zhan, Jin, Zhang, & Li, 2017) and miR‐29a (Li, Wang, Li, & Jing, 2017). Therefore, the identification of other miRNAs involved in the development of SCLC represents an opportunity to improve the therapeutic outcome in patients.

In this study, we assessed the chemoresistance effect of *SOX2* amplification in DMS114 cells under cisplatin treatment. Then, the differential expression of miRNA levels in two independent cohorts were analyzed between cells with *SOX2* overexpression and normal expression. Finally transcriptome, bioinformatics, and quantitative real‐time PCR (qPCR) analysis further investigated the relationship of downregulated miRNAs and *SOX2*.

## 2 METHODS AND MATERIALS

### 2.1 Cell lines and cell culture

Human lung cancer cell line DMS114 was obtained from the American Type Culture Collection, and other human lung cancer cells (NCI‐H209, NCI‐H446, NCI‐H358, and A549) were obtained from the Cell Resource Center at the Institute of Basic Medical Sciences, Chinese Academy of Medical Sciences. All cells were cultured in Dulbecco's Modified Eagle Medium (DMEM; Gibco) supplemented with 10% fetal bovine serum (FBS; Gibco), 100 IU/ml penicillin, and 100 μg/ml streptomycin (all Gibco) in a humidified atmosphere containing 5% CO_2_ at 37°C and subcultured every 3 days at a confluence of 10%–30%.

### 2.2 Plasmid construction and cell transfection

Lentiviral transduction of *SOX2* cDNA was performed by transfecting DMS114 cells with a lentiviral vector encoding the *SOX2* cDNA (pSin SOX2; Addgene), together with the accessory plasmids Gagpol, VSVG, and RSV‐REV, as described (Karachaliou et al., 2013). Parallel transfections were performed with empty plasmids as a control. Viral supernatants from either *SOX2* cDNA or control transfections were used to infect the DMS114 cell lines stably transduced with either empty vector (LV003), or overexpression vector (*SOX2*), with a density of 5 × 10^5^ cells in 10‐cm plates. The vector was transfected into cells with packaging plasmids using lipofectamine 2000 (Thermo Fisher Scientific) according to the manufacturer's protocol. After 48 hr, the supernatant was filtered and used for virus transduction into cells with 5 µM polybrene (Sigma‐Aldrich). Stable clones were obtained after selection by puromycin (Gibco).

### 2.3 Half‐maximal inhibitory concentration (IC_50_) analysis

Cisplatin was purchased from Aladdin. The DMS114 cells were collected before and after overexpression of *SOX2* to make a single cell suspension with a concentration of 5 × 10^4^ cells using DMEM containing 10% FBS (Gibco). Then, 96‐well plates were inoculated according to different final concentrations of cisplatin (0, 1.25, 2.5, 5, 10, 20, 40, 80 and 160 μM), and the final volume of each well was 100 μl. Three replicates were performed for each group. The cells were incubated for 48 hr at 37°C in 5% CO_2_ and saturated humidity. Then 10 μl of MTS solution was added, and incubation was continued for 2 hr. Finally, the absorbance value at 492 nm was determined, and the IC_50_ was calculated separately.

### 2.4 Small RNA library construction and high‐throughput sequencing

The DMS114 cells both before and after *SOX2* overexpression were separately extracted for total RNA, including the small RNA, fraction using Trizol reagent (Invitrogen). The quality and quantity of the isolated RNA were determined by using an ND‐1000 Nanodrop UV‐Vis spectrophotometer (Thermo Fisher Scientific), while RNA integrity was evaluated using the Agilent 2200 TapeStation (Agilent Technologies) using an RNA integrity number equivalent >7.0. Two small RNA libraries were constructed and sequenced using Illumina TruSeq deep sequencing technology and a TruSeq^®^ Small RNA Sample Prep Kit (Illumina) according to the manufacturer's instructions. Briefly the RNAs were ligated with a 3ʹ RNA adapter, followed by a 5ʹ adapter ligation. Subsequently, the adapter‐ligated RNAs were subjected to reverse transcription‐PCR and amplified with a low number of cycles. Then, the PCR products were size‐selected by polyacrylamide gel electrophoresis (PAGE). The purified library products were evaluated using the Agilent 2200 TapeStation and diluted to 10 p.m. for cluster generation in situ on the HiSeq 2500 single‐end flow cell, followed by sequencing (1#x00D7;50 bp) on a HiSeq 2500 platform. The image files generated by the sequencer were processed to produce digital quality data (raw FASTQ files).

### 2.5 Bioinformatic analysis

The resulting raw data were filtered to generate clean reads (18–30 nt) and then annotated by aligning to miRBase 21 (http://www.mirbase.org) to obtain known miRNAs. The expression profiles of known miRNAs were identified and compared. Significant differentially expressed miRNA changes were selected based on the following criteria: (a) statistical significance: miRNA expression changes were identified using a false discovery rate threshold of 0.05; and (b) fold change expression: a minimum two‐fold difference in either direction was required. The miRNA expression was normalized as the number of transcripts per million (TPM) clean tags as follows: TPM = (number of transcripts mapping to miRNA/ number of transcripts in clean data) × 10^6^.

#### 2.5.1 Target gene prediction for differentially expressed miRNAs

miRanda 3.3a software was employed to predict target genes for differentially expressed miRNAs.

#### 2.5.2 Gene ontology (GO) and kyoto encyclopedia of genes and genomes (KEGG) pathway analysis

The putative genes were subjected to GO enrichment and KEGG pathway analysis with DAVID 6.7 software (https://david.ncifcrf.gov/). Fisher's exact test and the χ^2^ test were used to select the significant GO categories and signaling pathways. The threshold of significance was defined by the P value, with *p* < .01 regarded as significant for both the GO and KEGG analysis.

#### 2.5.3 miRNA‐target genes network analysis

Cytoscape 3.7.2 software (https://cytoscape.org/) was used to map a network between the miRNAs and their target genes.

### 2.6 qPCR analysis

A total of 0.5–1 μg of total RNA prepared from the DMS114 cells before and after *SOX2* overexpression were reverse transcribed using a TaqMan microRNA Reverse Transcription Kit (Applied Biosystems) with Human Pool A and Human Pool B Megaplex RT Primers (Applied Biosystems). The reverse transcription reaction conditions were thermally cycled under the following conditions: 30 min at 16°C, 30 min at 42°C, and 5 min at 85°C. The products were stored at −20°C for later use or immediately processed according to the manufacturer's protocol. qPCR was performed in 96‐well reaction plates with an ABI 7500 Fast Real‐Time PCR System (Applied Biosystems). Each reaction was performed in a 20 μl volume system containing 1 μl of TaqMan small RNA assay, 1.33 μl of product from reverse transcription, 10 μl of TaqMan Universal PCR Master Mix (no AmpErase UNG) and 7.67 μl of nuclease‐free water. Template‐free controls were used to evaluate background signal. The qPCR program consisted of incubation at 50°C for 2 min, 95°C for 10 min, followed by 40 cycles each of denaturation at 95°C for 15 s and annealing and extension for 60 s at 60°C. The mean expression level of human endogenous control (*β‐actin*) was used as an internal control in all miRNA experiments to allow for the comparison of expression results. Each sample was run in three duplicates and relative quantification of miRNA expression was calculated using the 2^−ΔΔCt^ method.

### 2.7 Western blot (WB) analysis

Cells or tissue samples were lysed in RIPA buffer with protease inhibitors, centrifuged at 12,000 rpm for 15 min, and the supernatant was collected. The protein lysates (30 μg) were separated by sodium dodecyl sulfate‐PAGE and transferred to a polyvinylidene difluoride membrane. After blocking, the membrane was incubated with anti‐human polyclonal antibodies against *SOX2* (sc‐365964; Santa Cruz Biotechnology) and *β‐actin* (Sigma‐Aldrich), followed by incubation with a secondary antibody, mouse IgG HRP‐conjugated antibody (HAF007, R&D Systems, Inc.) and visualization using the ECL Detection System (GE Healthcare).

## 3 RESULTS

### 3.1 Differential expression of *SOX2* in lung cancer cells

**Figure 1:**
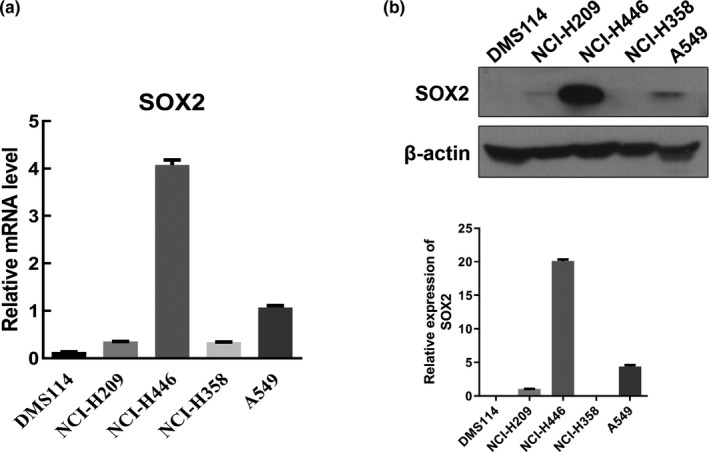
qPCR and WB detection of background expression of *SOX2* in five different lung cancer cell lines (DMS114, NCI‐H209, NCI‐H446, NCI‐H358, and A549). (a) Relative mRNA expression levels of *SOX2* in five different lung cancer cell lines by qPCR; (b) WB detection of relative *SOX2* protein expression levels

*SOX2* expression levels were estimated and quantified in five commonly used lung cancer cell lines. qPCR analysis showed that *SOX2* mRNA levels were significantly the highest in NCI‐H446 cells compared with the other lung cancer cell lines, and of *SOX2* expression was the lowest in DMS114 cells (Figure 1a). The protein expression of *SOX2* were also verified and confirmed to be the same using WB analysis (Figure 1b). Because we wanted to investigate the effect of changes in *SOX2* expression in SCLC, the DMS114 cells with the lowest level of *SOX2* expression were selected for use in further experiments of cisplatin resistance.

### 3.2 Overexpression of *SOX2* in lung cancer cells resulted in enhanced cisplatin resistance

**Figure 2:**
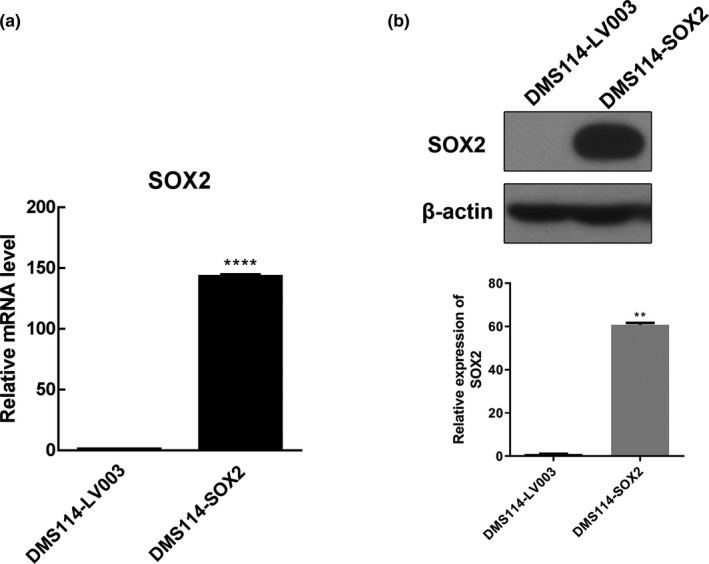
Overexpression of *SOX2* in DMS114 verified by qPCR and WB. (a) DMS114 cells were treated with the vector overexpressing *SOX2* and the blank control vector, and the relative mRNA expression levels of *SOX2* was determined by qPCR. (b) Relative *SOX2* protein expression was determined by WB analysis in the cells treated with the overexpressing vector and the negative control cells

DMS114 cells were transfected with lentiviral vectors expressing *SOX2*, and the cells were used for experiments after culturing in a drug‐free medium for 2 months. *SOX2* expression was verified by qPCR (Figure 2a) and WB (Figure 2b) in both DMS114 cells transfected with the *SOX2* overexpression vector and negative control cells transfected with an empty vector. The *SOX2* mRNA and protein levels were significantly higher in the DMS114 cells transfected with *SOX2* overexpression vector compared with the negative control cells transfected with the empty vector (Figure 2), which was accepted as proof that the DMS114 cells transfected with *SOX2* overexpression vector were constructed successfully.

**Figure 3:**
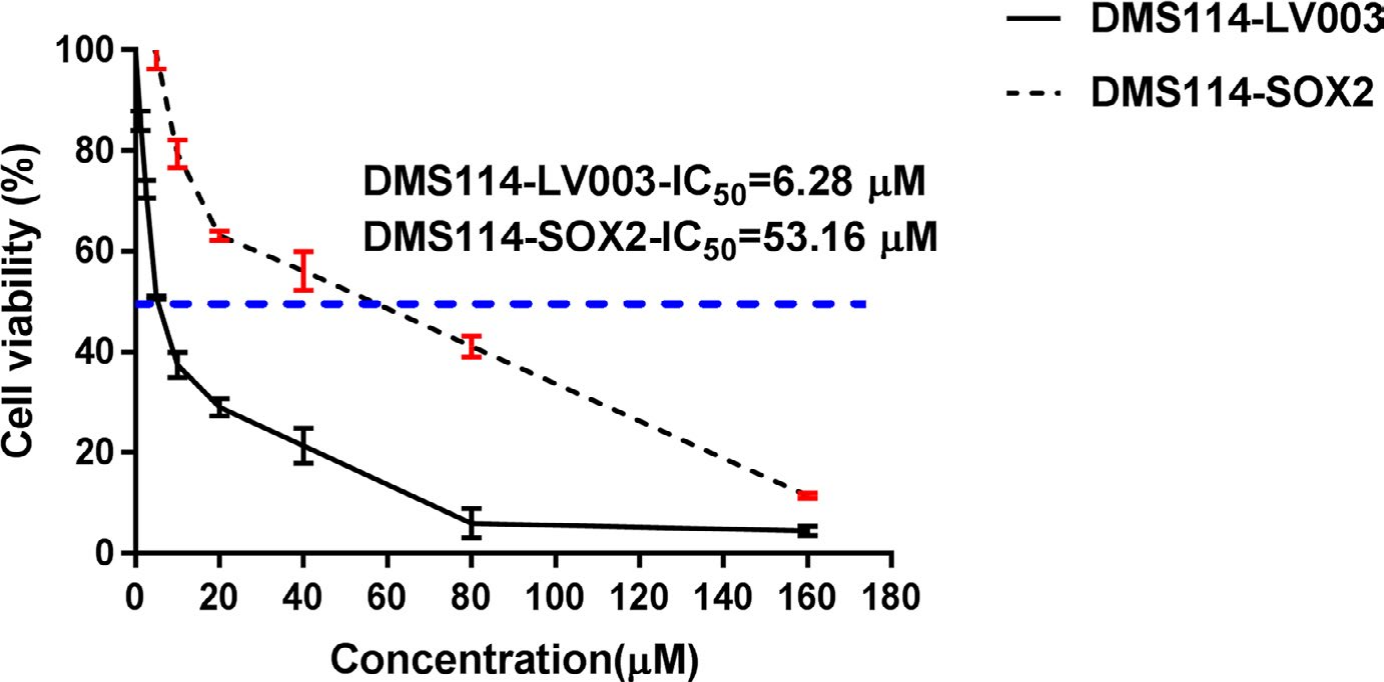
MTS analysis of the *SOX2*‐overexpressing cells and control cells under cisplatin resistance

Then, we determined the IC_50_ value of the *SOX2*‐overexpressing cells and control cells under cisplatin resistance using MTS analysis. The IC_50_ of *SOX2*‐overexpressing cells was higher than that in normal lung cancer cells (Figure 3). This suggested that the chemoresistance of *SOX2*‐overexpressing cells was indeed higher than the control cells and that the overexpression of *SOX2* was positively correlated with cisplatin resistance. Thus, it was necessary to further determine if miRNAs could affect the expression of *SOX2* in the *SOX2*‐overexpressing cells in cisplatin resistance.

### 3.3 High‐throughput sequencing of differential miRNA expression between DMS114 cells before and after *SOX2* overexpression

**Figure 4:**
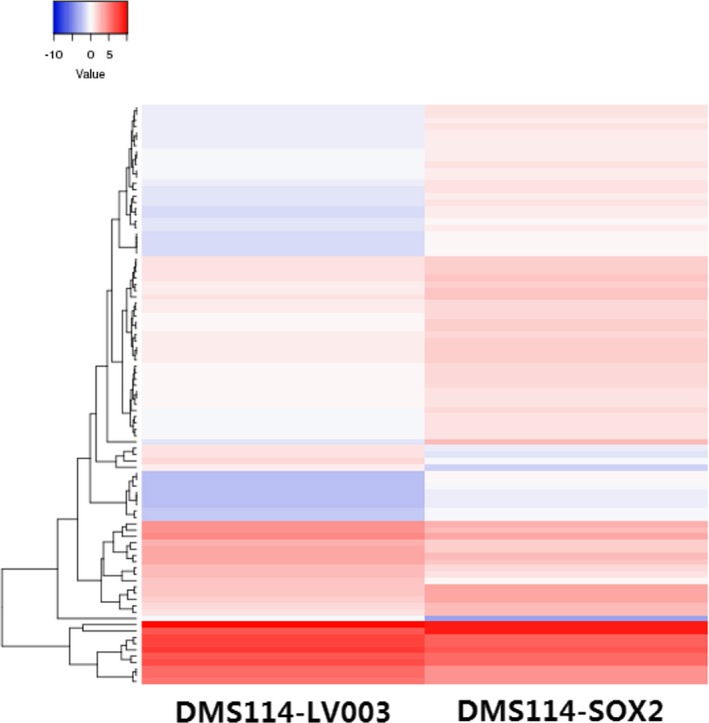
Differential heat map of miRNA expression before and after *SOX2* overexpression of in DMS114 cells

High‐throughput sequencing analysis was performed to screen the differentially expressed miRNAs between the *SOX2*‐overexpressing cells and the control cells, which revealed that 68 miRNAs were upregulated and 24 miRNAs were downregulated (Figure 4). The 24 downregulated miRNAs were further verified and are listed in Table 1.

**Table 1:** 24 downregulated miRNAs in the *SOX2*‐overexpressing cells

miRNA ID	DMS114‐LV003‐TPM	DMS114‐*SOX2*‐TPM	*p*‐value	FDR
hsa‐miR‐518e‐3p	9.795E−01	7.732E−02	5.452E−03	3.678E−02
hsa‐miR‐4477a	1.539E + 00	3.093E−01	7.233E−03	4.471E−02
hsa‐miR‐372‐3p	5.107E + 00	1.237E + 00	5.053E−06	8.498E−05
hsa‐miR‐6873‐3p	2.169E + 00	5.412E−01	4.188E−03	2.939E−02
hsa‐miR‐6773‐3p	2.148E + 01	5.799E + 00	1.284E−18	7.561E−17
hsa‐miR‐877‐3p	2.239E + 00	6.186E−01	6.570E−03	4.161E−02
hsa‐miR‐410‐5p	1.091E + 01	3.247E + 00	5.978E−09	1.683E−07
hsa‐miR‐6774‐5p	2.938E + 00	9.278E−01	4.794E−03	3.311E−02
hsa‐miR‐335‐3p	5.345E + 01	2.072E + 01	2.078E−23	1.682E−21
hsa‐miR‐7‐1‐3p	1.891E + 02	7.400E + 01	6.933E−77	2.565E−74
hsa‐miR‐3182	5.373E + 01	2.103E + 01	5.165E−23	3.822E−21
hsa‐miR‐369‐3p	1.474E + 02	5.791E + 01	9.594E−60	2.259E−57
hsa‐miR‐188‐5p	5.597E + 00	2.242E + 00	2.231E−03	1.756E−02
hsa‐miR‐4791	1.967E + 02	8.389E + 01	9.802E−64	2.539E−61
hsa‐miR‐301a‐5p	8.885E + 00	3.866E + 00	5.644E−04	5.516E−03
hsa‐miR‐4448	5.597E + 00	2.474E + 00	7.914E−03	4.778E−02
hsa‐miR‐32‐5p	2.659E + 01	1.183E + 01	6.842E−09	1.906E−07
hsa‐miR‐7‐5p	1.178E + 02	5.250E + 01	3.104E−34	4.232E−32
hsa‐miR‐4419b	4.604E + 01	2.057E + 01	3.295E−14	1.446E−12
hsa‐miR‐3529‐3p	1.164E + 03	5.429E + 02	7.487E−277	4.848E−274
hsa‐miR‐548aj‐5p	1.028E + 01	4.949E + 00	1.761E−03	1.462E−02
hsa‐miR‐1299	1.140E + 01	5.567E + 00	1.352E−03	1.152E−02
hsa‐miR‐130b‐5p	1.735E + 01	8.583E + 00	1.137E−04	1.339E−03
hsa‐miR‐340‐5p	2.355E + 02	1.174E + 02	8.241E−45	1.334E−42

Note

DMS114‐LV003‐TPM: miRNA expression in DMS114 cells transfected with the empty vector; DMS114‐*SOX2*‐TPM: miRNA expression in DMS114 cells transfected with *SOX2* overexpression vector.

### 3.4 Function analysis of differentially expressed miRNAs’ target genes

**Figure 5:**
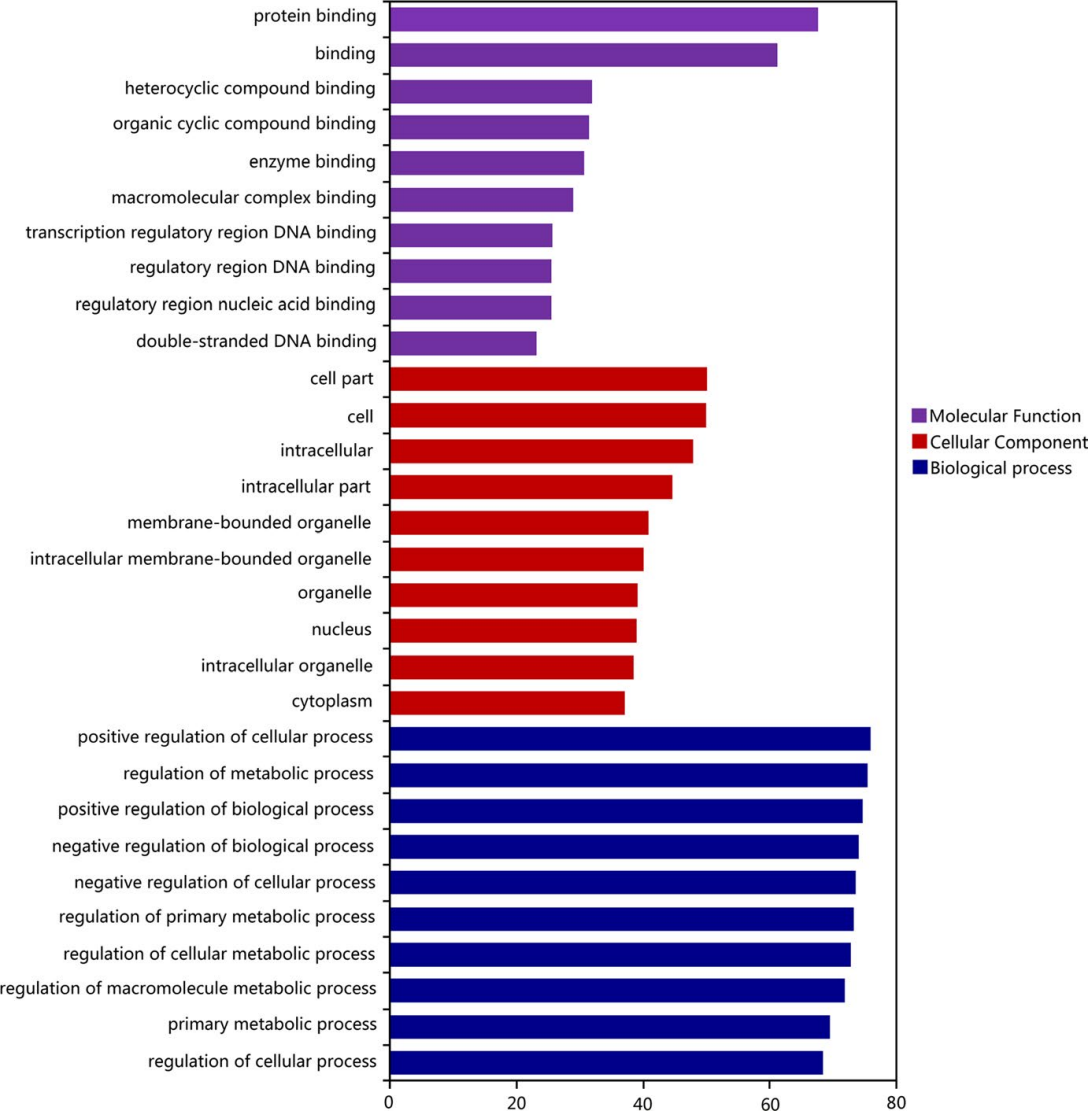
GO analysis of target genes for differentially expressed miRNAs. The GO analysis identified three ontologies, which described the molecular function of the gene, the cellular component, and the biological process

To understand the function of the identified downregulated miRNAs, GO and KEGG analysis were performed to analyze their target genes. The GO analysis showed that the target genes of differentially expressed miRNAs were enriched in various biological process and molecular functions. Target genes for differentially expressed miRNAs were mainly concentrated in the biological processes (Figure 5).

**Figure 6:**
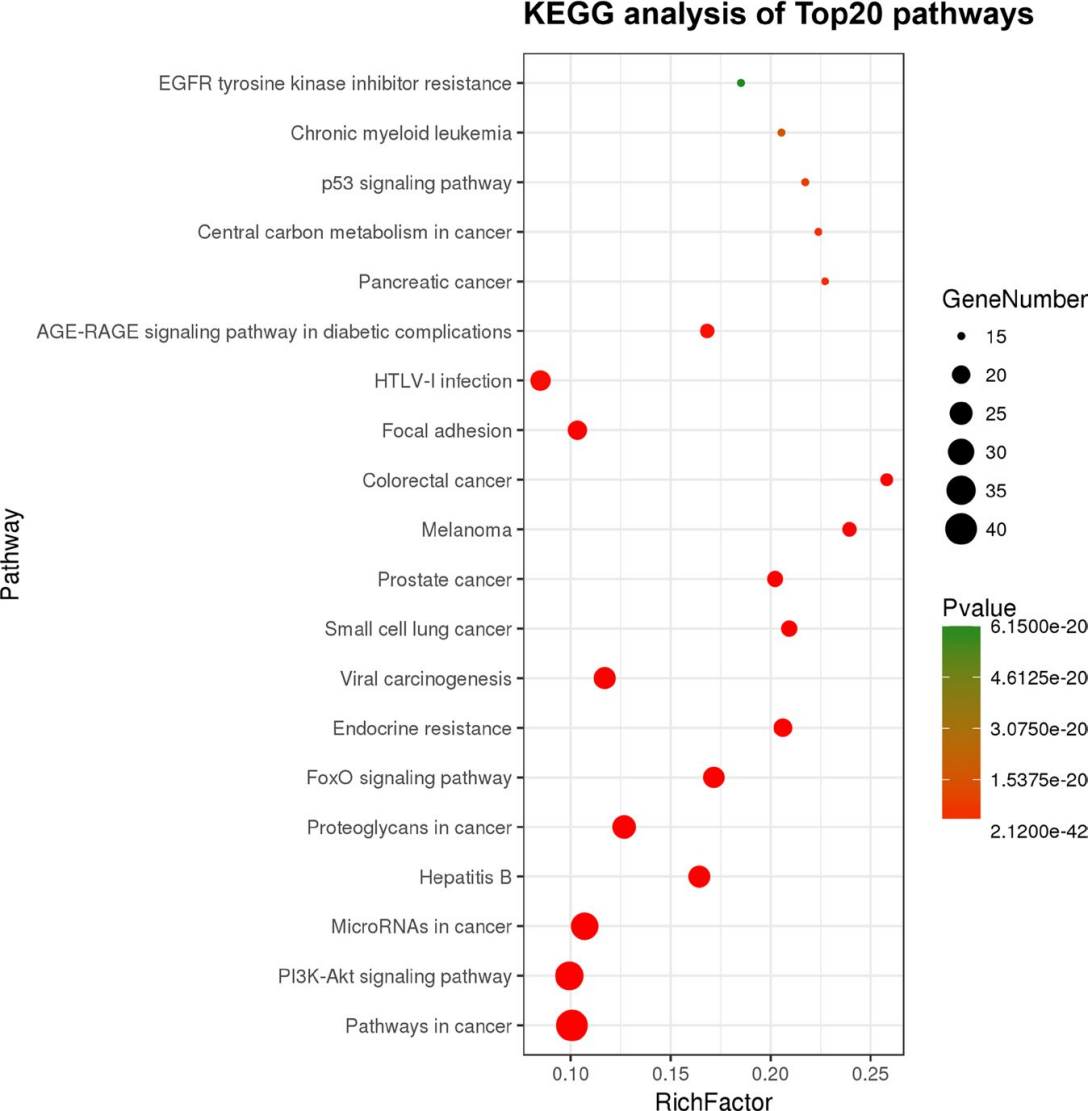
KEGG analysis of Top20 pathways. The ordinate represents the pathway entry, and the abscissa represents the rich factor (the ratio of the number of target genes annotated to the pathway term to the number of genes annotated to the pathway entry). The point represents the number of significant target genes, and the size of the point represents a significant target. The higher the number of genes, the larger the point and the greater the number of genes with significant targets

Genes usually play a role in biological functions by interacting with each other. KEGG pathway analysis aids in understanding more about the biological functions of genes and is used to analyze the metabolic pathway enrichment of target genes. The analysis showed that the downregulated miRNAs' target genes were involved in pathways in cancer, the PI3K‐AKT signaling pathway, and microRNAs in cancer (Figure 6). There were some genes also involved in the platinum drug resistance pathway (Figure S1).

### 3.5 Network regulation map showing the downregulated miRNAs associated with *SOX2* binding

**Figure 7:**
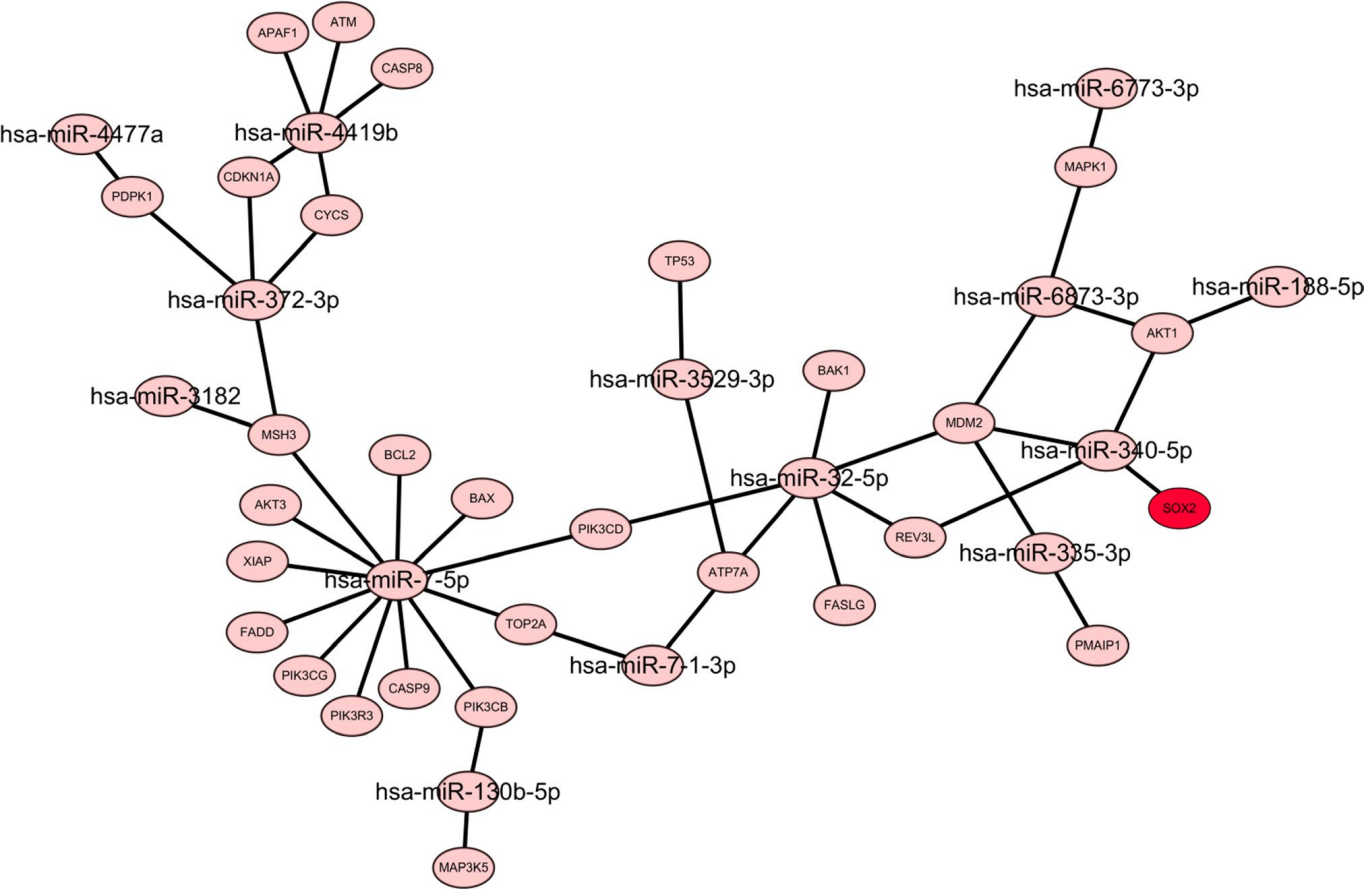
Network regulation map of downregulated miRNAs, *SOX2*, and other genes in the platinum drug resistance pathway

**Figure 8:**
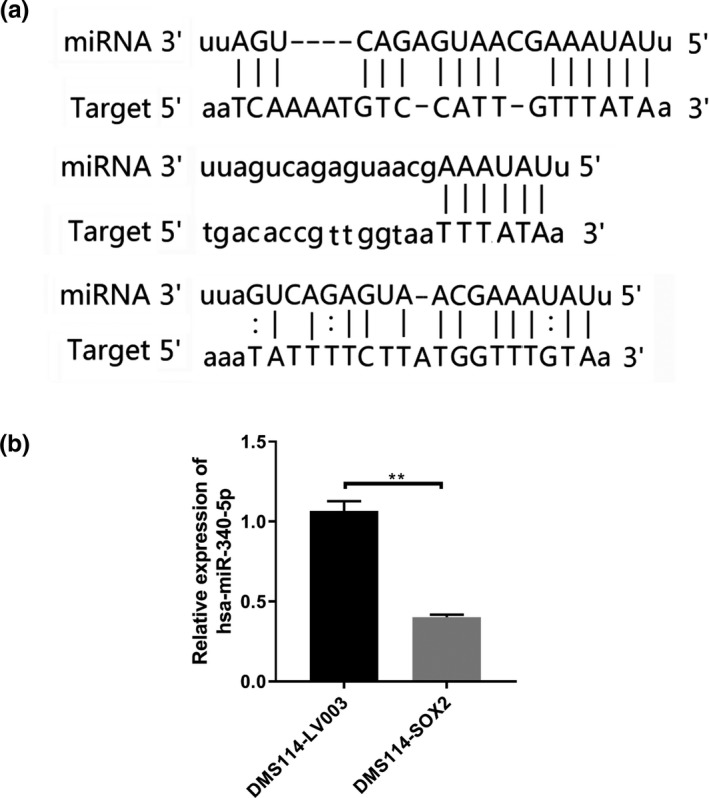
The detailed relationship between hsa‐miR340‐5p and *SOX2*. (a) The detailed binding location of hsa‐miR‐340‐5p and *SOX2* by miRanda analysis. (b) The expression of hsa‐miR340‐5p was verified by qPCR before and after *SOX2* overexpression DMS114 cells

Network regulation mapping of the downregulated miRNAs, *SOX2*, and other genes from platinum drug resistance pathway showed that only 14 downregulated miRNAs could target *SOX2* and other genes from platinum drug resistance pathway, while only one miRNA, hsa‐miR‐340‐5p, was able to bind with *SOX2* (Figure 7). In the miRanda analysis, hsa‐miR‐340‐5p showed a higher binding affinity with *SOX2* for three locations (Figure 8a). Hence, we concluded that hsa‐miR‐340‐5p might interact with *SOX2* to mediate cisplatin resistance in SCLC by regulating its expression.

### 3.6 Identification of hsa‐miR‐340‐5p by qPCR

To prove the above hypothesis, the expression of hsa‐miR‐340‐5p was verified by qPCR. The results showed that hsa‐miR‐340‐5p expression was significantly decreased in the *SOX2*‐overexpression cells compared with the normal cells (Figure 8b). Thus, the qPCR verified that hsa‐miR‐340‐5p could indeed interact with *SOX2* by regulating its expression.

## 4 DISCUSSION

SCLC is a highly metastatic lung cancer subtype, accounting for up to 20% of all lung cancer cases worldwide. The conventional treatment options for SCLC with cisplatin have remained unchanged despite the development of drug resistance and increased mortality with a reduced overall median survival rate (Tripathi et al., 2017; Umemura et al., 2014). Even though SCLC management has followed the major developments of modern cancer treatment through the integration of biology, imaging, chemotherapy, and radiotherapy, the prognostic improvement and treatment has remained substantially the same for the past 25 years (Karachaliou et al., 2013; Rosell & Wannesson, 2012).

Recent studies have also found that *SOX2* is highly expressed in a variety of tumors, such as lung cancers like NSCLC (Leung et al., 2010; Lu et al., 2010) and SCLC (Rudin et al., 2012), pancreatic cancer (Herreros‐Villanueva, Bujanda, Billadeau, & Zhang, 2014), gastric carcinoma (Li et al., 2004), breast cancer (Lengerke et al., 2011), glioblastomas (Gangemi et al., 2009), osteosarcomas (Basu‐Roy et al., 2012), prostate cancer (Bae et al., 2010), and hepatocellular carcinomas (Xu et al., 2010). *SOX2* could activate important gene cascades for all these cancers that may affect a variety of functions, such as tumor proliferation, migration, and drug resistance. However, the exact role of *SOX2* in mediating SCLC progression of in lung cancer patients has not been fully elucidated due to the limited accessibility of patient tissues for research purposes (Tripathi et al., 2017). It was previously reported that amplification and overexpression of *SOX2* were strongly associated with SCC morphology and its favorable clinicopathological features (Karachaliou et al., 2013). In contrast, these features were less frequent in SCLC and were rare in adenocarcinoma and of uncertain prognostic significance. Hence, we assumed that the elucidation of *SOX2*‐dependent pathways may identify novel therapeutic vulnerabilities in SCLC for the management of tumor regression.

An immunohistochemical analysis of *SOX2* expression in various lung cancer types found that SCLC tissues expressed a higher level of *SOX2* than NSCLC tissues (Karachaliou et al., 2013). In parallel, *SOX2* was found to cooperate with important oncogenes, like *Wnt1* (OMIM: 164820), *Wnt2* (OMIM: 147870), *c‐Myc* (OMIM: 190080), and *Notch1* (OMIM: 190198), to promote lung tumor occurrence, while downregulation of *SOX2* inhibited proliferation and induced apoptosis in tumor cells (Chen et al., 2012). These findings were consistent with our results of *SOX2* expression in SCLC cell lines. To assess the relevance of *SOX2* in SCLC, we analyzed a panel of SCLC cell lines for *SOX2* mRNA and protein expression and selected the lowest expression of *SOX2* cells to be used in our experiments with the overexpression of *SOX2* by lentiviral vector‐mediated transfection. Our results were consistent with previous reports on the amplification of *SOX2* and the characterization of its role as an oncogene in lung and esophageal SCC (Bass et al., 2009). A high level of *SOX2* protein expression and elevated gene copy numbers have been reported in SCLC and in lung and esophageal SCCs, which indicate that *SOX2* is an important oncogene and confirms its identity as a genuine SCLC driver gene (Rudin et al., 2012).

Many studies have recently reported and evaluated the oncogenic potential of tumor‐suppressive miRNAs in different subtypes of lung cancers (Guan et al., 2012; Li et al., 2018, 2017; Wang et al., 2017). Several studies have reported that resistance to cisplatin and other anticancer drugs are mediated through miRNAs targeting the expression of different oncogenes in many cancers. Yang et al. reported that miR‐214 induced cell survival and cisplatin resistance by targeting *PTEN* (OMIM: 601728) in ovarian cancer (Yang et al., 2008). Another study by Zhu et al. reported that the miRNA cluster miR‐200bc/429 was promoted apoptosis by targeting B‐cell lymphoma 2 and X‐linked inhibitor of apoptosis protein, which sensitized resistant lines to vincristine as well as to cisplatin in gastric and lung cell lines (Zhu et al., 2012). Imanaka et al. found that miR‐141 was the most highly expressed miRNA in cisplatin‐resistant cell lines and was ectopically expressed in cisplatin‐sensitive cell lines, directly targeting the 3ʹ untranslated region of yes‐associated protein 1 (*YAP1*, OMIM: 606608), which is known to play an important role in apoptosis induced by DNA‐damaging agents. Thus, downregulation of *YAP1* expression by overexpressing miR‐141 renders cells more resistant to DNA‐damaging drugs (Imanaka et al., 2011). Ye et al. have reported that miR‐376c overexpression blocked cisplatin‐induced cell death, whereas siRNA anti‐miR‐376c enhanced the effect of cisplatin (Ye et al., 2011).

We hypothesized that miRNAs might be involved in the development of cisplatin resistance in SCLC, which is mediated via the regulation of *SOX2* expression. We analyzed the differentially expressed miRNAs using high‐throughput sequencing of *SOX2*‐overexpression cells and compared the expression levels with that of normal cells. The analysis showed that 68 miRNAs were upregulated, and 24 miRNAs were downregulated in the *SOX2*‐overexpression cells. We further verified the 24 downregulated miRNAs, *SOX*2, and genes in the platinum drug resistance pathway using KEGG analysis by network analysis. Furthermore, the qPCR analysis also found that a cancer‐related miRNA, hsa‐miR‐340‐5p, which showed a higher binding affinity with *SOX2* under miRanda analysis, was markedly downregulated in *SOX2*‐ overexpression cells.

A better understanding of the genomic changes in SCLC is helpful to identify new therapeutic targets. However, a systematic genomic analysis of SCLC is difficult because this cancer subtype is rarely treated surgically, resulting in the lack of suitable tumor specimens for comprehensive analysis. Hence, further studies are necessary to fully elucidate the involvement of miRNA‐mediated overexpression of oncogenic factors in SCLC in drug resistance and to find a powerful tool to cure lung cancers.

## 5 CONCLUSION

In conclusion, our report might be the first direct investigation of the relationship between the downregulated expression of hsa‐miR‐340‐5p with the overexpression of *SOX2* in cisplatin‐resistant SCLC cells. Hence, the observations of this study indicate that *SOX2*‐mediated cisplatin resistance in SCLC could be affected by downregulating hsa‐miR‐340‐5p expression. A more detailed elucidation of the mechanism using in vivo experiments to investigate the involvement of *SOX2* in mediating cisplatin resistance in SCLC is expected to be clarified in the future.

## CONFLICT OF INTEREST

The authors declare that they have no conflict of interest.
