# Supplementary figures and images for: SOX2 mediates cisplatin resistance in small‐cell lung cancer with downregulated expression of hsa‐miR‐340‐5p
Source: Mol Genet Genomic Med. 2020 Mar 4;8(5):e1195. doi: 10.1002/mgg3.1195 (PMC7216814; doi:10.1002/mgg3.1195)

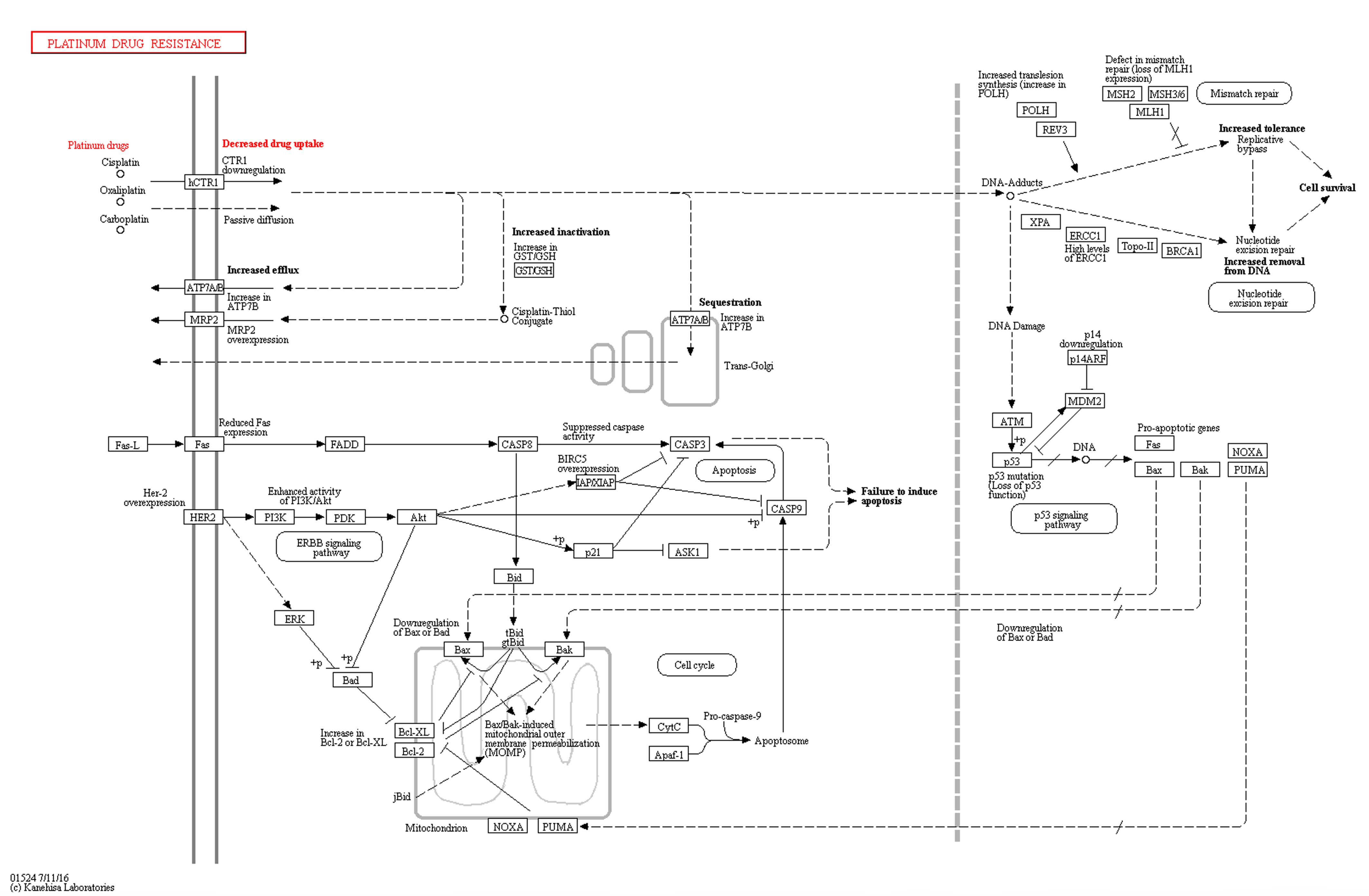

Supplement: Supplementary file 1 [file MGG3-8-e1195-s001.tif]
